# Supplementary material for: Proteomics-based receptor-ligand matching enhances differentiation maturity of human-stem-cell-derived neurons
Source: Stem Cell Reports. 2025 Aug 21;20(9):102604. doi: 10.1016/j.stemcr.2025.102604 (PMC12447311; doi:10.1016/j.stemcr.2025.102604)
Supplement: Document S1. Figures S1 and S2 [file mmc1.pdf]

Stem Cell Reports, Volume *20*

Supplemental Information

Proteomics-based receptor-ligand matching enhances differentiation maturity of human-stem-cell-derived neurons

Dimitar Dimitrov, Yi Lien, Tetsuya Hori, Yukiko Goda, Christian Rosenmund, and Zacharie Taoufiq

Proteomics-based Receptor-ligand Matching Enhances Differentiation Maturity of Human Stem Cell-derived Neurons

SUPPLEMENTAL INFORMATION

Figure S1: Distinct Synaptosomal UD Proteome Profiles of NGN2-Neurons Versus Mammalian Whole Brain.
(A) Electron microscopy of iN synaptosomal fraction showing intact synaptic components, such as the active zone (AZ) and synaptic vesicles (SVs).
(B) Immunoblot analysis of iN subcellular fractionation showing significant purity of iN synaptosomes (P2) from the enrichment of synaptic proteins.
C) Comparison of synaptic protein families (synaptotagmins, syntaxins, active zone and postsynaptic density proteins) identified by HD vs UD proteomics.
(D) Numbers of identified proteins in iN synaptosomes by HD vs UD proteomics.
(E) Quantitative UD proteomic comparison of synaptic machinery in iN synaptosomes versus mammalian whole brain purified synaptosomes. Bar graphs represent normalized iBAQ ratios of proteins (mean ratio ± SEM, n = 3; p-value: ***< 0.001).
(F) Schematic diagram showing major differences in the synaptosomal proteome between NGN2-induced neurons from hiPSCs and those from the mammalian (rat) whole brain. NGN2 synapses are depicted as 100% glutamatergic due to the incomplete and low expression of key inhibitory synapse machinery such as VGAT.

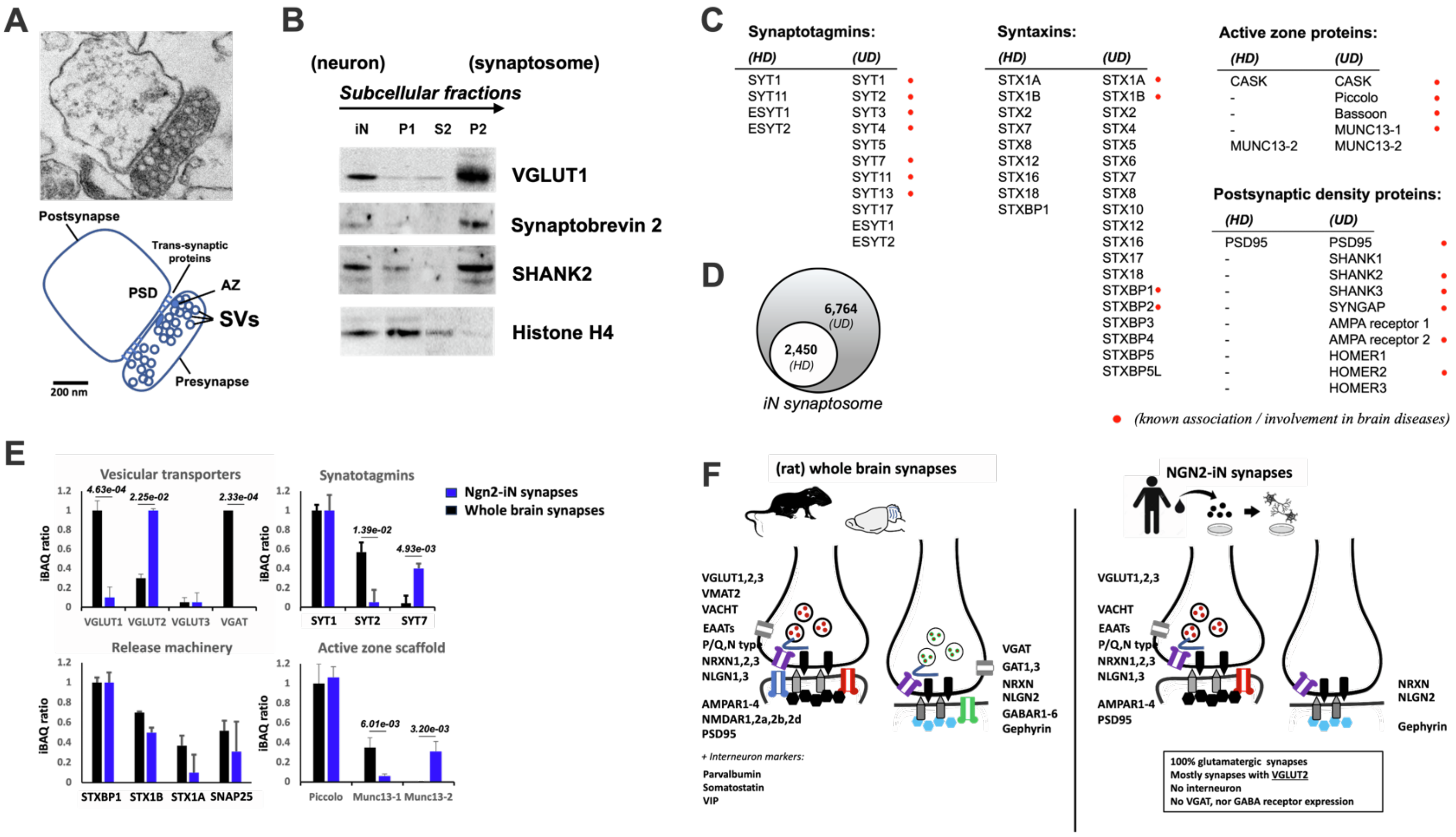

Figure S2: Transcriptomic Analysis of BN vs. BCGNr iN Cells Reveals Upregulation of Neuronal Structural and Immune Stress-Resistance Genes in BCGNr Neurons.

(A) For normalization of BN and BCGNr cell whole transcriptomes, eight astrocytic genes and one iPSC gene were selected as reference genes, covering a broad range of expression levels, as their expression is not expected to change under our culture conditions. The scaling factor was derived from the geometric mean of their expression. Scatter plots and correlation coefficients are shown before and after correction and normalization.

(B) Heatmap showing fold changes in mRNA expression in BCGNr vs BN iN cells. BCGNr cells show higher expression of synaptic (e.g., *SYP, SYN1, GRIA1*), structural (e.g., *TUBB3, MAP2, MAPT*), and neuronal activation markers (*FOS, EGR1*), while astrocytic and iPSC-related genes (e.g., *GFAP, GJA1, SOX2*) remain unchanged or slightly lower than in BN cells (cell differentiation batches n = 2).

(C) Identification of the most strongly differentially expressed genes between BN and BCGNr iN cells. In BCGNr, *DTX3L, SAMD9*, and IFI6 showed 800-, 180-, and 100-fold higher transcript counts, respectively. Conversely, *AMIGO3, 7SK_5*, and *OPRK1* were highly downregulated in BCGNr compared to BN iN cells by 1000-, 300-, and 15-fold (cell differentiation batches n = 2).

(D) Table summarizing the most strongly differentially expressed genes and their associated functions. Genes upregulated in BCGNr cells remain unstudied in neurons but are associated with cell survival, stress resistance, and suppression of mitochondrial stress-related during immune responses. In contrast, genes upregulated in BN cells are well-characterized in neurons and are associated with inhibition of axon and neurite growth, delayed differentiation, and altered neuronal excitability.

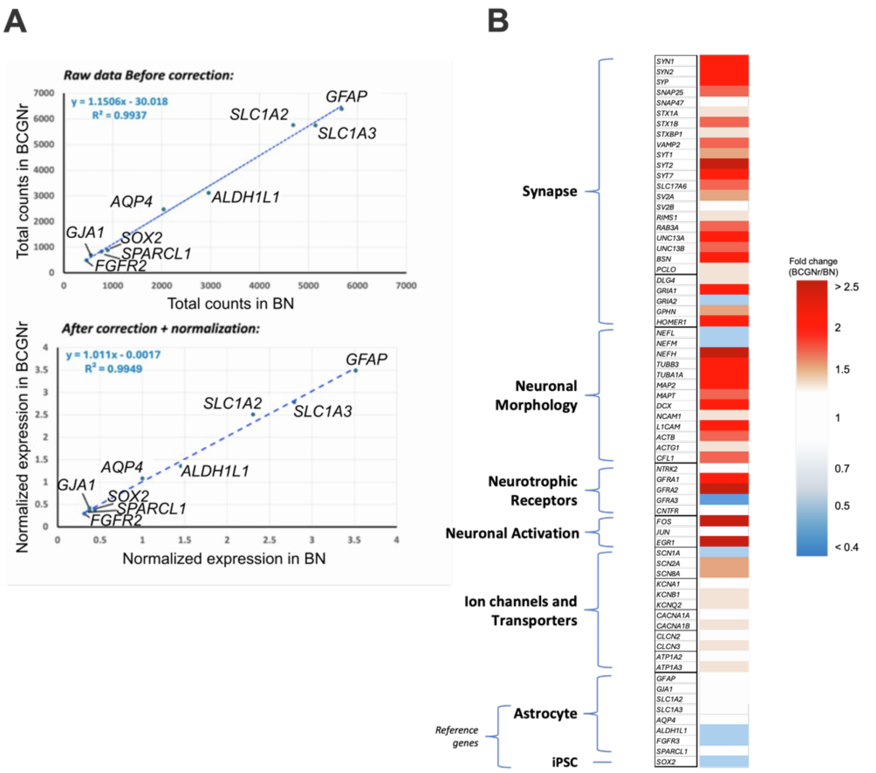

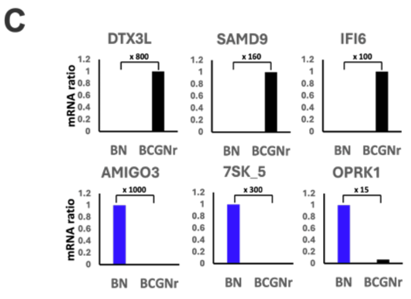

D

Most strongly differentially expressed genes & funcitons

In BCGNr/BN cells:

		references (cell functions)	direct role in neurons?
DTX3L	promotes **cell survival** by enhancing DNA repair, modulating **immune responses**, and increasing **stress resistance**	Saleh *et al., J Mol Biol* 2024; Yang *et al., Mol Cell* 2017	*uncharacterized*
SAMD9	serves as a **protective factor under stress**, particularly in **immune responses** and cellular **damage control**	Hou *et al., Nat Comm* 2025; Zhang et al., Science Adv 2023	*uncharacterized*
IFI6	supports cell survival by promoting **mitochondrial stability**, reducing ROS production, and **dampening** excessive **inflammation**	Cheriyath *et al., J Clin Invest* 2007; Liu *et al., J Exp Clin Can Res* 2020	*uncharacterized*

In BN/BCGNr cells:

AMIGO3	**inhibits axon regeneration and neurite outgrowth**, with elevated expression linked to regenerative failure following injury	Ahmed *et al., PlosOne* 2013; Almutiri *et al., Sci Reports* 2018	*Yes*
7SK_5	inhibits transcription elongation, contributes to **cell cycle pausing** and a**ltered differentiation**, under stressful conditions	Nguyen et al., Nature 2001; Liu *et al., Cell* 2013	*Yes*
OPRK1	**reduces neurite outgrowth** and branching, leads to **decreased neuronal excitability** and neurotransmitter release	Snyder *et al., Neuron* 2018; Reeves *et al., Front Mol Neuro* 2022	*Yes*

Supplemental References:
(Related to Figure S2D)

Ahmed, Z., Douglas, M.R., John, G., Berry, M., and Logan, A. (2013). AMIGO3 is an NgR1/p75 co-receptor signalling axon growth inhibition in the acute phase of adult central nervous system injury. PLoS One *8*, e61878. 10.1371/journal.pone.0061878.

Almutiri, S., Berry, M., Logan, A., and Ahmed, Z. (2018). Non-viral-mediated suppression of AMIGO3 promotes disinhibited NT3-mediated regeneration of spinal cord dorsal column axons. Sci Rep *8*, 10707. 10.1038/s41598-018-29124-z.

Cheriyath, V., Glaser, K.B., Waring, J.F., Baz, R., Hussein, M.A., and Borden, E.C. (2007). G1P3, an IFN-induced survival factor, antagonizes TRAIL-induced apoptosis in human myeloma cells. J Clin Invest *117*, 3107-3117. 10.1172/JCI31122.

Hou, G., Beatty, W., Ren, L., Ooi, Y.S., Son, J., Zhu, Y., Sheng, Q., Huang, W., Li, D., Liu, C., et al. (2025). SAMD9 senses cytosolic double-stranded nucleic acids in epithelial and mesenchymal cells to induce antiviral immunity. Nat Commun *16*, 3756. 10.1038/s41467-025-59090-w.

Liu, W., Ma, Q., Wong, K., Li, W., Ohgi, K., Zhang, J., Aggarwal, A., and Rosenfeld, M.G. (2013). Brd4 and JMJD6-associated anti-pause enhancers in regulation of transcriptional pause release. Cell *155*, 1581-1595. 10.1016/j.cell.2013.10.056.

Liu, Z., Gu, S., Lu, T., Wu, K., Li, L., Dong, C., and Zhou, Y. (2020). IFI6 depletion inhibits esophageal squamous cell carcinoma progression through reactive oxygen species accumulation via mitochondrial dysfunction and endoplasmic reticulum stress. J Exp Clin Cancer Res *39*, 144. 10.1186/s13046-020-01646-3.

Nguyen, V.T., Kiss, T., Michels, A.A., and Bensaude, O. (2001). 7SK small nuclear RNA binds to and inhibits the activity of CDK9/cyclin T complexes. Nature *414*, 322-325. 10.1038/35104581.

Reeves, K.C., Shah, N., Munoz, B., and Atwood, B.K. (2022). Opioid Receptor-Mediated Regulation of Neurotransmission in the Brain. Front Mol Neurosci *15*, 919773. 10.3389/fnmol.2022.919773.

Saleh, H., Liloglou, T., Rigden, D.J., Parsons, J.L., and Grundy, G.J. (2024). KH-like Domains in PARP9/DTX3L and PARP14 Coordinate Protein-Protein Interactions to Promote Cancer Cell Survival. J Mol Biol *436*, 168434. 10.1016/j.jmb.2023.168434.

Snyder, L.M., Chiang, M.C., Loeza-Alcocer, E., Omori, Y., Hachisuka, J., Sheahan, T.D., Gale, J.R., Adelman, P.C., Sypek, E.I., Fulton, S.A., et al. (2018). Kappa Opioid Receptor Distribution and Function in Primary Afferents. Neuron *99*, 1274-1288 e1276. 10.1016/j.neuron.2018.08.044.

Yang, C.S., Jividen, K., Spencer, A., Dworak, N., Ni, L., Oostdyk, L.T., Chatterjee, M., Kusmider, B., Reon, B., Parlak, M., et al. (2017). Ubiquitin Modification by the E3 Ligase/ADP-Ribosyltransferase Dtx3L/Parp9. Mol Cell *66*, 503-516 e505. 10.1016/j.molcel.2017.04.028.

Zhang, F., Ji, Q., Chaturvedi, J., Morales, M., Mao, Y., Meng, X., Dong, L., Deng, J., Qian, S.B., and Xiang, Y. (2023). Human SAMD9 is a poxvirus-activatable anticodon nuclease inhibiting codon-specific protein synthesis. Sci Adv *9*, eadh8502. 10.1126/sciadv.adh8502.
