# Supplementary material for: Proteomics-based receptor-ligand matching enhances differentiation maturity of human-stem-cell-derived neurons
Source: Stem Cell Reports. 2025 Aug 21;20(9):102604. doi: 10.1016/j.stemcr.2025.102604 (PMC12447311; doi:10.1016/j.stemcr.2025.102604)
Supplement: Document S2. Article plus supplemental information [file mmc3.pdf]

Stem Cell Reports

ISSCR

Report

OPEN ACCESS

Proteomics-based receptor-ligand matching enhances differentiation maturity of human-stem-cell-derived neurons

Dimitar Dimitrov,[1,*] Yi Lien,[2] Tetsuya Hori,[1] Yukiko Goda,[1] Christian Rosenmund,[2] and Zacharie Taoufiq[1,3,*]

[1]Synapse Biology Unit, Okinawa Institute of Science and Technology Graduate University, 1919-1, Tancha, Onna-son, Okinawa 904-0495, Japan

[2]Institut für Neurophysiologie, Charité Universitaetsmedizin Berlin, Corporate Member of Freie Universität Berlin, Humboldt-Universität zu Berlin, Berlin Institute of Health, 10117 Berlin, Germany

[3]Lead contact

*Correspondence: ddimitrov@oist.jp (D.D.), zacharie.taoufiq@oist.jp (Z.T.)

https://doi.org/10.1016/j.stemcr.2025.102604

SUMMARY

Human-induced pluripotent stem cell (hiPSC) technology enables generation of various cell types, offering significant potential for regenerative medicine and personalized disease modeling. However, optimizing the functional maturity of differentiated cells is crucial for improving their reliability in research. Here, we introduce a deep-proteomics-based “receptor-ligand matching” (RLM) strategy to inventory surface receptors on differentiated cells and adjust the culture conditions accordingly. Focusing on an NGN2-induced neuron (iN) model, which rapidly produces glutamatergic neurons but exhibits modest synaptic activity, we identified 3,934 iN membrane proteins, including dozens of growth factor receptors and, notably, the complete GDNF receptor family (GFRA1, GFRA2, and GFRA3) previously undetected. Supplementing culture media with selected ligands enhanced neuronal health, neurite density, and synaptogenesis. Electrophysiology measurements confirmed greater functional synaptic maturity and responsiveness in optimized iNs compared to conventionally generated iNs. The RLM strategy offers a versatile approach to enhance the health and functionality of potentially any hiPSC-derived cell type.

INTRODUCTION

Human-induced pluripotent stem cell (hiPSC) technology offers immense opportunities in biomedical research, enabling repairing treatment development through cell differentiation and tissue engineering. This process reprograms somatic cells to a pluripotent state by expressing transcription factors like OCT4, SOX2, KLF4, and MYC (Takahashi and Yamanaka 2006). hiPSCs can then be expanded indefinitely, stored, and differentiated into various cell types (neural, cardiac, skin, liver, blood, and pancreatic) (Shi et al., 2017; Rowe and Daley 2019). While initial reprogramming and differentiation are well studied, optimizing differentiated cells has received less focus. Yet, enhancing cell health and maturity is crucial for improving iPSC-derived cell utility, reproducibility, and experimental consistency, as well as reducing research time and costs. Therefore, optimizing iPSC-derived cells is key to fully harness the potential of personalized and regenerative medicine.

For instance, forced expression of NGN2 can rapidly convert iPSCs to neurons (induced neurons [iNs]), generating a nearly pure glutamatergic population (Zhang et al., 2013). Due to this efficiency, the NGN2-iN model is frequently used in brain disease studies, such as schizophrenia, Alzheimer disease, and amyotrophic lateral sclerosis (Huang et al., 2017; Li et al., 2021; Giacomelli et al., 2022). However, NGN2 neurons often exhibit reduced extent of synaptic maturation and activity especially at early stages (Zhang et al., 2013; Hulme et al., 2022), which could be due to their rapid differentiation, which limits full synaptic proteome development. Without extended culture periods (>6 weeks) (Gunhanlar et al., 2018; Burlingham et al., 2022; Bullmann et al., 2024), NGN2 neurons lack robust activity associated with reduced branching networks and responsiveness in patch-clamp recordings, an important limitation for applications in disease modeling and drug screening.

In this study, we present a versatile proteomics-based strategy, termed “receptor-ligand matching” (RLM), to optimize the widely used NGN2-iN model. Applying our deep proteomics workflow, previously shown to uncover hidden proteomes in complex samples like brain synapses (Taoufiq et al., 2020), we analyze NGN2-neurons’ membrane protein composition. The plasma membrane proteome with its surface receptors can help reveal essential extracellular ligands and signaling needs of a cell. We identified and quantified 3,934 membrane proteins, including 29 growth factor receptors, 15 of which are known to be neurotrophic. Among these, 10 showed $\geq$10-fold higher expression in induced neurons compared to iPSCs—some of which are undetectable by conventional proteomics, such as the complete GDNF-receptor family (GFRA1, 2, and 3). By targeting these receptors, we supplemented the medium with relevant ligands and assessed the RLM-based effects. In both mass and autaptic cultures, NGN2 neurons showed improved health, neurite density, and synaptogenesis maturation, leading to greater responsiveness and

Published by Elsevier Inc. on behalf of International Society for Stem Cell Research.
This is an open access article under the CC BY license (http://creativecommons.org/licenses/by/4.0/).

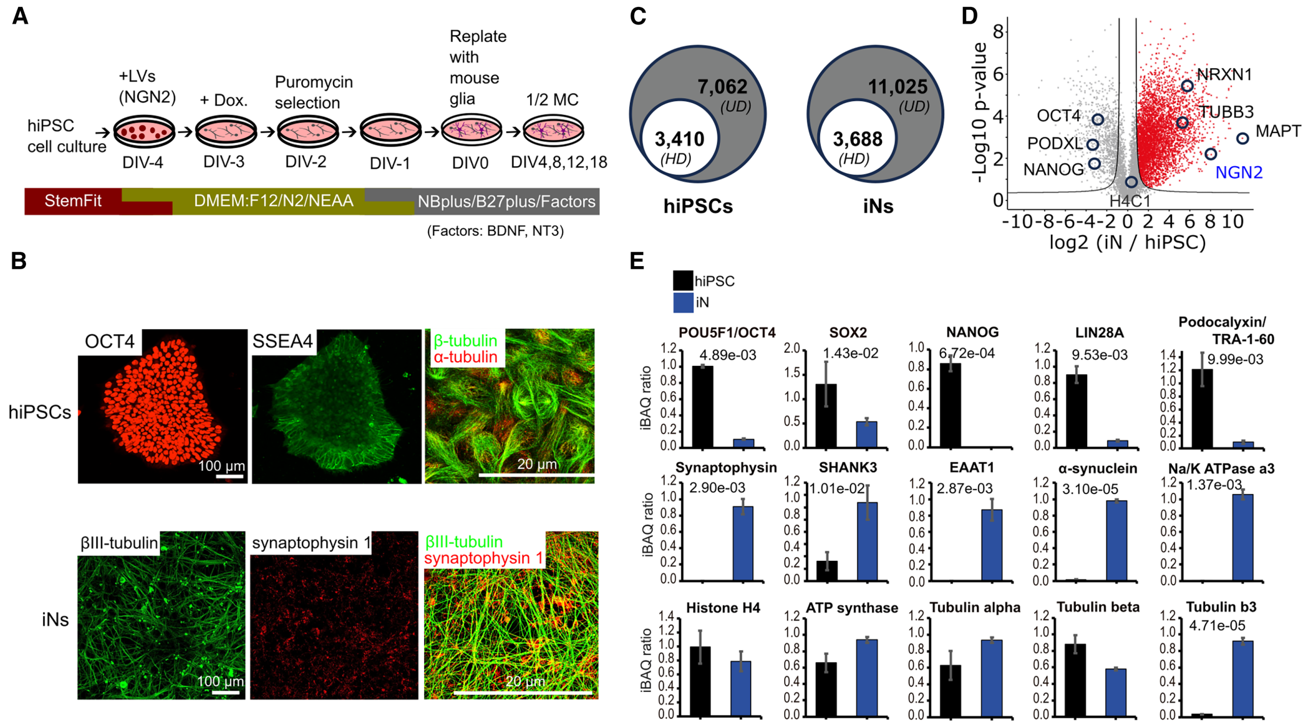

Figure 1. Comprehensive proteomic profiling of hiPSCs differentiation efficiency into neurons

(A) Schematic of the iN-differentiation protocol. After replating with mouse glia, half of the neurobasal medium was changed and supplemented with BDNF and NT3 (50 ng/mL) every 4 days.

(B) Immunofluorescence images of hiPSCs (HPS0331; pluripotency markers: Oct4, Ssea4) and iNs at DIV18 (neurites: beta-3 tubulin; synapses: synaptophysin).

(C) Identified protein numbers in hiPSCs and iNs using UD versus HD Proteomics.

(D) Volcano plot illustrating proteomic changes from hiPSCs to NGN2-induced neurons at 18 days. *x* axis ($\log_2$ scale): mean iN/hiPSC normalized label-free iBAQ ratio. *y* axis (log10 scale): *p* value from three separate batches. Genes encoding specific proteins are labeled; NGN2 (genetically overexpressed) is shown in blue.

(E) Quantitative UD proteomics comparison of pluripotency, neuronal, and housekeeping markers in hiPSCs vs. iNs. Bar graphs show normalized iBAQ ratios (mean ± SEM, $n = 3$ separate batches; *p* values indicated).

increased average amplitude to stimuli as measured by electrophysiology.

RESULTS

UD proteomics unveils hidden proteomes and measures hiPSC differentiation efficiency into NGN2-neurons

We used conventional single-step induction by overexpressing NGN2 to convert human iPSCs into functional neurons (iNs) (Zhang et al., 2013). As shown in Figure 1A, lentiviral delivery for NGN2 expression, combined with a mouse glial/neurobasal medium with added BDNF and NT3, supports iPSC differentiation into iNs over 18 days. To assess differentiation efficiency, we conducted immunofluorescence imaging on hiPSCs and iNs at DIV18. Figure 1B illustrates hiPSCs expressing pluripotency markers OCT4 and SSEA4. Higher-resolution cytoskeletal imaging reveals hiPSCs as round-shaped, with some in division. After 18 days, iNs display neuronal morphology, with branched axons, dendrites (beta-3 tubulin), and synapses (synaptophysin).

We then used proteomics to examine protein changes during hiPSC differentiation into neurons, applying our "ultra-definition" (UD) workflow, previously shown to uncover hidden proteomes in brain synapses (Taoufiq et al., 2020). This method optimizes protein digestion and peptide separation compared to "high-definition (HD) proteomics," which uses one-step protein digestion and high-definition mass spectrometry device. UD proteomics doubled/tripled protein identifications in hiPSCs/iNs compared to HD proteomics (Figure 1C). It also identified three times more proteins in purified iN synaptosomes, including previously undetected synaptic proteins. UD proteomics revealed notable proteome differences between NGN2-neuron synaptosomes and those from

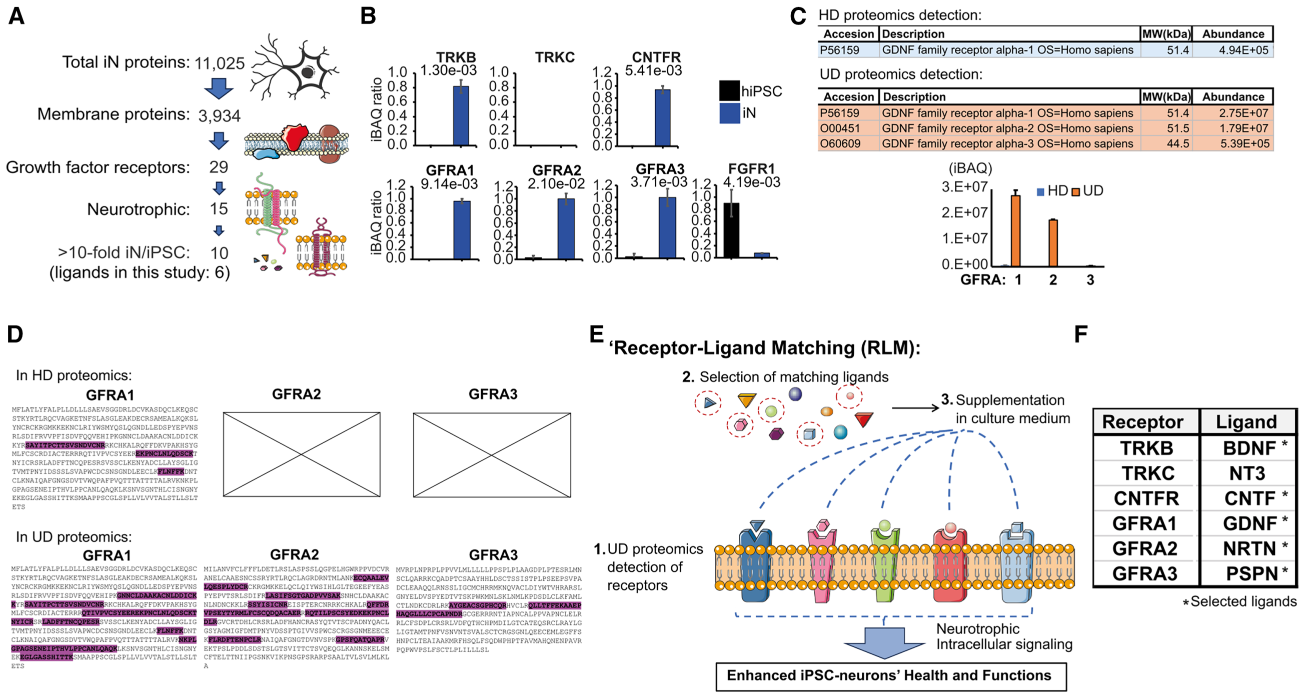

Accesion	Description	MW(kDa)	Abundance
P56159	GDNF family receptor alpha-1 OS=Homo sapiens	51.4	4.94E+05

Accesion	Description	MW(kDa)	Abundance
P56159	GDNF family receptor alpha-1 OS=Homo sapiens	51.4	2.75E+07
O00451	GDNF family receptor alpha-2 OS=Homo sapiens	51.5	1.79E+07
O60609	GDNF family receptor alpha-3 OS=Homo sapiens	44.5	5.39E+05

Receptor	Ligand
TRKB	BDNF *
TRKC	NT3
CNTFR	CNTF *
GFRA1	GDNF *
GFRA2	NRTN *
GFRA3	PSPN *

Figure 2. Optimization of NGN2-neuron differentiation medium using UD proteomics data

(A) iN proteome data mining using UniProt Knowledgebase to identify the selected membrane receptors for the study.

(B) Growth factor receptor expression in hiPSCs versus iNs. Bar graphs show normalized label-free iBAQ ratios (mean ± SEM, $n = 3$ separate HPS0331 batches; p values indicated).

(C) UD versus HD proteomics comparison of the GDNF receptor family detection in iNs. Includes UniProt ID, receptor nomenclature, molecular weight (MW), and mean iBAQ values (mean ± SEM, $n = 3$ separate batches).

(D) Unique peptide coverage of the GDNF receptor family, highlighting amino acid sequences, detected by HD and UD proteomics.

(E) Schematic of the receptor-ligand matching (RLM) principle.

(F) RLM table for neurotrophic factor receptors in iNs. Ligands selected for subsequent experiments are marked with an asterisk (*), based on UD proteomics results.

whole mammalian brain (Figure S1). As shown in Figure 1D, the shift in proteome from hiPSCs to iNs at 18 days confirms effective differentiation, with key neuronal proteins (e.g., neurexin-1, TUBB3, and MAPT) appearing. Quantitative analysis further showed high pluripotency markers (OCT4, SOX2, and LIN28A) in hiPSCs, whereas neuronal and synaptic proteins (synaptophysin, SHANK2, and α-synuclein) were prominent in iNs but nearly absent in hiPSCs (Figure 1E). Control housekeeping proteins (e.g., ATP synthase and tubulin) were similarly expressed in both cell types. These results underscore UD proteomics' depth and its label-free quantification reliability in studying human stem cell and neuronal proteomes.

UD proteomics drives the optimization of NGN2-neuron differentiation culture medium

The plasma membrane, housing cell receptors, is crucial for cell function and health. Understanding its receptor composition can guide improvements in cell culture by adding specific cues for differentiated cells. Using UD proteomics data, we analyzed the hiPSC and iN cell surface proteomes. We first leveraged the newly unveiled UD proteome information to explore the hiPSC and iN cell surface proteomes. By cross-referencing our data with UniProt annotations, we identified 11,025 proteins in iNs, including 3,934 membrane-localized proteins and 29 growth factor receptors. Among these, 10 known neurotrophic factor receptors were highly expressed (>10-fold) in iNs compared to hiPSCs, and for half of them, we tested the corresponding ligands (Figure 2A; supplemental "iN vs. iPS proteome resource," Table S1). UD proteomic quantifications revealed that neurotrophic receptors TRKB, CNTFR, and GDNFR-alpha (GFRA) 1, 2, and 3 were predominantly expressed in iNs. Conversely, FGFR1, a key regulator of pluripotency, was abundant in hiPSCs but low in iNs. Interestingly, TRKC, the NT3 receptor, was either undetected or too low for quantification in both cell types, despite NT3's common use in NGN2-neuron differentiation (Figure 2B).

We then analyzed the LC-MS/MS detection of the GDNF receptor alpha family. Only UD proteomics detected unique

peptide signatures for all GFRA family members, while traditional HD proteomics identified only GFRA1, at levels 50 times lower in iBAQ than UD proteomics (Figures 2C and 2D). This discrepancy may explain why GDNF receptor ligands are often omitted in NGN2-neuron culture supplements. Thus, we introduced the "receptor-ligand matching" (RLM) approach, using UD proteomics data to inventory receptors on hiPSC-differentiated cell surfaces (Figure 2E). This enables us to add specific ligands to the culture medium for TrKB, CNTFR, GFRA1, GFRA2, and GFRA3 receptors—namely, BDNF, CNTF, GDNF, neurturin (NRTN), and persephin (PSPN) (Figure 2F)—to potentially enhance the health and function of hiPSC-derived cells.

RLM-based factor combination enhances NGN2-neuronal health and synaptogenesis

To assess the impact of the new RLM-based factor combination compared to the conventional BDNF-NT3 on NGN2-induced neuron health and function, we first measured synaptic protein expression. Immunoblot analyses showed that cells without factors had the lowest expression of the presynaptic protein VGLUT1 and the postsynaptic protein SHANK2. The BDNF-NT3 combination increased both, while the RLM-based factors further enhanced synaptic protein expression, with the greatest effect seen with the BDNF-CNTF-GDNF-PSPN-NRTN combination. PSPN, however, showed minimal contribution to this increase (Figures 3A and 3B). Thus, we identified BDNF-CNTF-GDNF-NRTN [BCGNr] as the most effective combination for further experiments. This increase in synaptic proteins was accompanied by more mature synaptic structures in BCGNr-cultured iNs compared to BDNF-NT3 (BN), as evidenced by overlapping immunofluorescence signals of active zone proteins RIM1/2, Piccolo, and synaptophysin (Figures 3C and 3D). Morphological analysis also showed a significant increase in neurite network density in BCGNr-cultured iNs versus BN-cultured cells (Figures 3E and 3F). Transcriptomic analysis further confirmed a shift in the gene expression profile of BCGNr neurons toward enhanced stress resilience and neuronal development. This was evidenced by the downregulation of genes associated with inhibition of neuronal growth and the upregulation of immune response genes linked to cell survival and stress resistance in BCGNr versus BN iN cells (Figure S2; Table S2). Altogether, BCGNr supplementation markedly enhances iN cell health and synaptogenesis in NGN2-differentiated hiPSCs.

RLM-based factors increase physiological responsiveness of NGN2 neurons in both mass and autaptic culture models

To assess the impact of the RLM-based factor combination BCGNr on synaptic function, we performed electrophysiology on two hiPSC-derived neuronal culture models. First, we conducted paired recording stimulation on iNs in mass cultures (Figure 4A), yielding evoked excitatory postsynaptic current (EPSC) responses. Using the AMPA/kainate receptor inhibitor CNQX confirmed that these responses were excitatory (Figure 4B). In iNs cultured with BDNF-NT3, the response success rate at DIV19 was 31%, which increased to 93% with BCGNr (Figure 4C). While the paired pulse ratio, indicating presynaptic release efficacy, remained unchanged, cells cultured with BCGNr showed a significantly higher average EPSC amplitude compared to BDNF-NT3 cultures (Figure 4D).

We further evaluated the RLM-based factor combination BCGNr using iPSC-derived neurons in an autaptic culture system, as this model has become a standard for studying synaptic properties with precise recordings in a simplified and controlled environment compared to mass culture (Fenske et al., 2019; Rhee et al., 2019) (Figure 4E). Morphological fluorescence imaging revealed that autaptic neurons cultured with BCGNr exhibited denser, more developed neurite networks at DIV49 and DIV59 than those with BDNF-NT3 (Figure 4F). Capacitance measurements, correlating with neuronal membrane surface area, confirmed this, showing significantly higher capacitance in BCGNr-cultured neurons (Figure 4G). BCGNr also yielded a higher response recording success rate (92%) compared to BDNF-NT3 (64%) and a significantly higher average EPSC amplitude (Figures 4H and 4I). Spontaneous synaptic activity, measured by miniature EPSCs (mEPSCs), showed significant increases in both frequency and amplitude with BCGNr, indicating enhanced synaptic strength and further demonstrating the BCGNr combination's improvement of iPSC-derived neuron health and synaptogenesis (Figure 4J).

DISCUSSION

In this study, we utilized deep proteomics to analyze membrane protein composition in the widely used NGN2-iN model, focusing on neurotrophic growth factor receptors. Among 3,934 membrane proteins identified, 29 growth factor receptors were expressed on the iN-cell surface, including 15 involved in neurotrophic signaling. Five of these receptors, with commercially available ligands, were highly more expressed in iNs than hiPSCs. Leveraging this insight, we optimized culture medium supplements to enhance hiPSC differentiation, producing neurons with enhanced health and functionality. We termed this approach "deep-proteomics-based RLM."

While most users of NGN2-iN models commonly use BDNF and NT3 in differentiation media (Zhang et al., 2013; Li et al., 2021; Hulme et al., 2022; Bullmann et al., 2024; Fenske et al., 2019), targeting TRKB and TRKC receptors, respectively, our data showed low TRKC levels, with

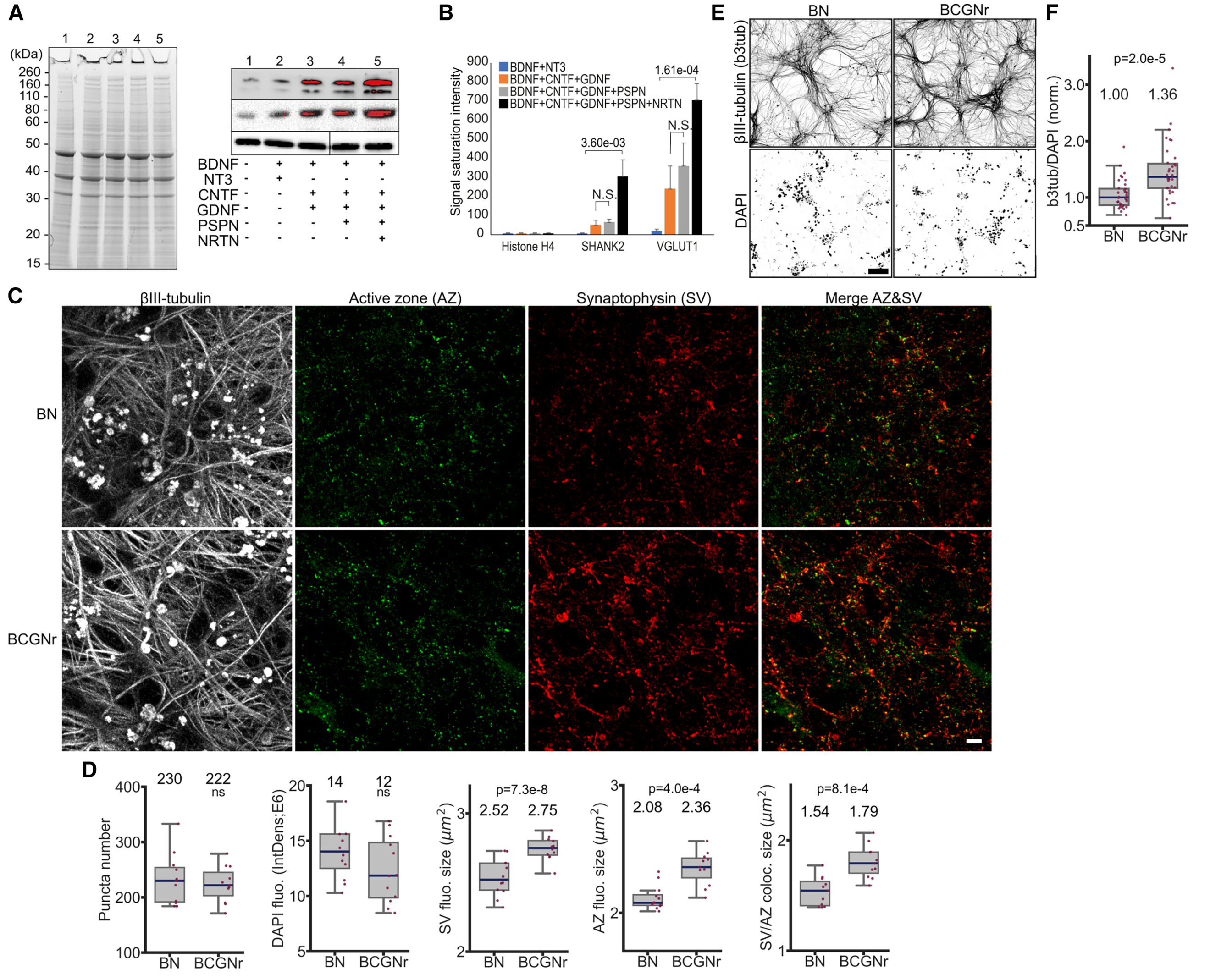

Figure 3. Impact of RLM-based factor combinations on synaptogenesis and neurite network density in NGN2 neurons

(A) Immunoblot analysis of synaptic protein expression in NGN2-induced neurons cultured with various neurotrophic factor combinations (50 ng/mL each). Left: SDS-PAGE gel staining. Right: immunoblots for synaptic markers Shank2 and VGLUT1, with Histone H4 as the loading control. Red pixels indicate signal saturation.

(B) Quantitative analysis of signal saturation (red pixels) intensities (mean ± SEM, $n = 3$ separate HPS0331 batches; N.S., not significant; p values indicated).

(C) Immunofluorescence imaging of active zone (AZ) and synaptic vesicle (SV) protein synaptophysin (scale bars, 10 μm).

(D) Quantification of AZ and SV fluorescence signal sizes, areas, and overlaps in BCGNr vs. BN cultured iNs (median and IQR; significance: p values indicated; $n = 4$ batches, 2 HPS0328 and 2 HPS0331).

(E) Morphological imaging of neurite network density (beta3-tubulin) in BCGNr-cultured iNs versus BN-cultured iNs (scale bars, 100 μm).

(F) Quantification of neurite density in BCGNr vs. BN cultured iNs labeled with beta3-tubulin and normalized to DAPI (cell nuclei) signal ($n = 36$, 4 batches, 2 HPS0328 and 2 HPS0331; median and IQR; significance: p values indicated).

TRKB highly expressed in NGN2 neurons. Consistently, removing NT3 had no effect on our neuronal differentiation, suggesting it may be unnecessary. Instead, we identified the optimal ligand combination BDNF, CNTF, GDNF, and NRTN using our RLM strategy. NRTN, the ligand for GFRA2 newly detected by our UD proteomics workflow (Taoufiq et al., 2020), was used here for the first time in NGN2-iNs differentiation. This novel combination likely enhanced neuronal survival and neurite outgrowth and indirectly promoted synaptogenesis. Indeed, electrophysiological assessments showed >90% cell responsiveness to patch-clamp stimuli, which is particularly advantageous for autaptic preparations

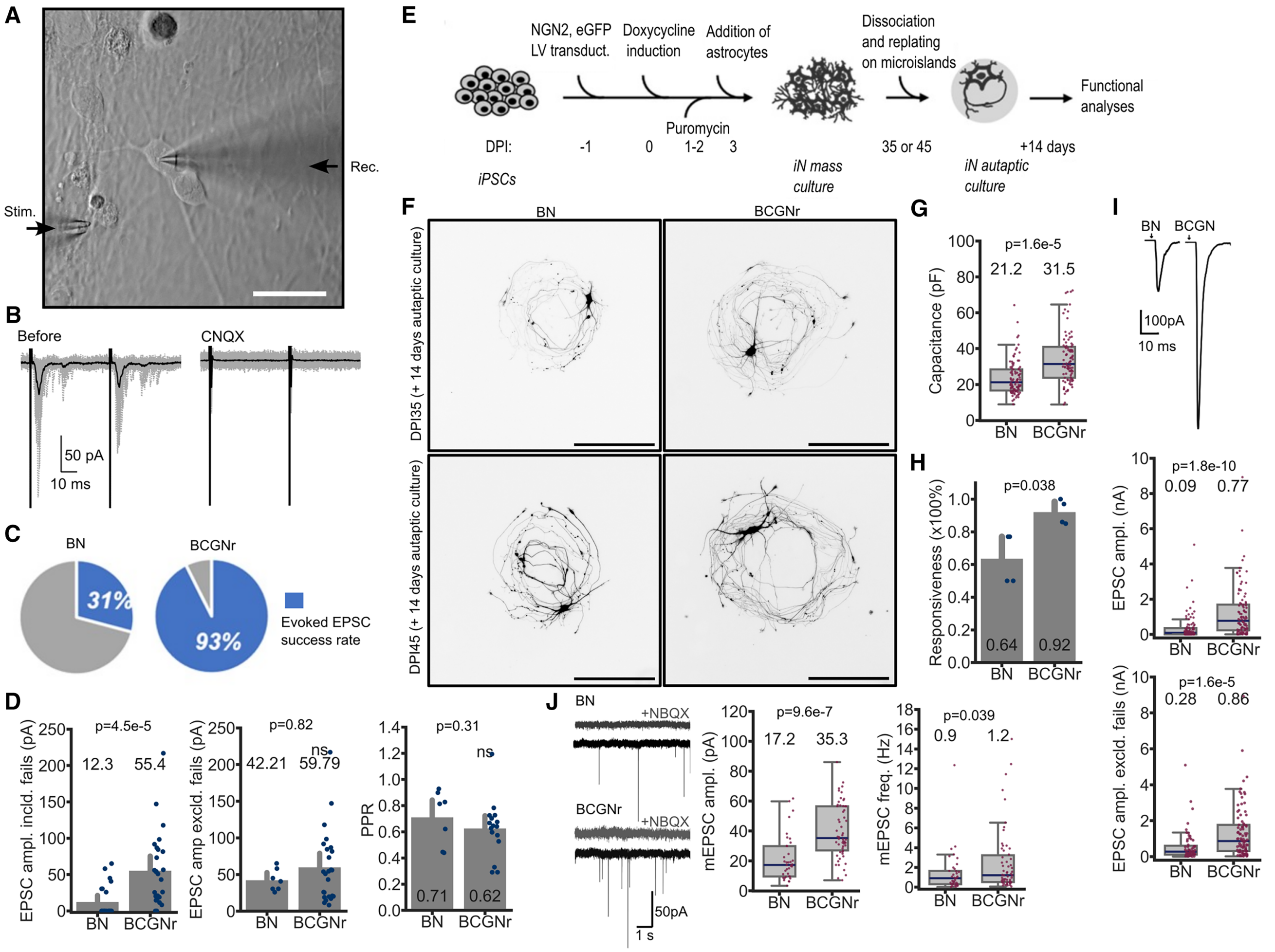

Figure 4. Enhanced neurophysiological function in hiPSC-derived neurons cultured with RLM-based factor combination

(A) Paired recording stimulation setup for (HPS0328) iN cells in mass cultures at DIV18 (DIC image, scale bars, 100 μm).

(B) Sample traces of evoked EPSCs before and after AMPA receptor antagonist CNQX application.

(C) Evoked EPSC responsiveness success and failure rates in iN cells cultured with BN vs. BCGNr.

(D) Mean amplitudes of evoked EPSCs in iNs cultured with BN including (left) and excluding (middle) response failures ($n = 24$) and BCGNr ($n = 27$). Right: mean paired pulse ratio in iNs cultured with BN ($n = 7$) and BCGNr ($n = 16$). Data were collected from three separate (HPS0328) iN batches; mean ± SEM. p values indicated; N.S., not significant.

(E) Schematic diagram for the autaptic iN culture from BIHi005-A iPSC line.

(F) Representative fluorescence images of DPI35 and DPI45 (+14 days) autaptic iNs cultured with medium supplemented with BN vs. BCGNr (scale bars, 50 μm).

(G) Average membrane capacitance of autaptic iNs with BN vs. BCGNr ($n = 94$ cells each, two separate batches of BIHi005-A).

(H) Percentage of cells with successful evoked EPSC recordings in autaptic iNs cultured with BN vs. BCGNr (58/92 BN and 89/96 BCGNr, two separate BIHi005-A batches).

(I) Representative traces (left) and means of evoked EPSC amplitude including (middle) and excluding (right) response failures in autaptic iNs cultured with BN ($n = 91$) vs. BCGNr ($n = 96$), two separate BIHi005-A batches.

(J) Representative traces (left), mean amplitude (center), and frequency (right) of miniature EPSCs recorded in autaptic iNs cultured with BN ($n = 37$) vs. BCGNr ($n = 55$), two separate BIHi005-A batches (p values indicated).

that typically require 50–70 days without a guaranteed high success rate of responsiveness.

To address the immaturity of NGN2-induced neurons, co-culture with mouse glia has been a common approach (Johnson et al., 2007; Zhang et al., 2013; Pasca et al., 2015). While astrocytes provide essential trophic support for neuronal survival, rodent astrocytes can introduce variability, affecting reproducibility and relevance of findings

in human disease models. Moreover, even when added in substantial numbers, astrocytes may not supply sufficient factors. To address this, serum-free supplements rich in antioxidants, vitamins, fatty acids, and added growth factors were developed to offer a more stable environment mimicking *in vivo* brain conditions and is widely used in neuron cultures (Chen et al., 2008; Zhang et al., 2013; Bardy et al., 2015). However, such optimizations are formulated for broad applications in neuronal models that differ in their membrane receptors. Here, we pursued a targeted approach to identify specific growth factors that robustly meet the NGN2-iN needs in culture.

Limitations arise from the results of our study using the mass culture system. Although we observed increased responsiveness to stimulation in BCGNr neurons, as well as clear structural differences in active zone morphology, these changes were not reflected in baseline synaptic measures such as EPSC amplitude or PPR in the 3-week iNs. One possibility is that the culture period was insufficient for the full functional integration of the enhanced structural features. Supporting this, our data from autaptic neurons revealed significantly increased evoked EPSC amplitudes. Additionally, the use of NGN2-induced neurons, which express a quantitatively distinct repertoire of presynaptic protein isoforms (e.g., VGLUT2 and MUNC13-2) compared to primary cortical neurons (VGLUT1 and MUNC13-1), may alter how structural changes in the AZ are functionally translated. Future studies incorporating longer culture durations, broader electrophysiological paradigms, and live imaging of synaptic vesicle dynamics will be essential to fully elucidate the relationship between presynaptic architecture and function in this model.

On another note, our synapse proteomics showed that NGN2-induced neurons lack VGAT and GABA receptors, despite expressing gephyrin and neuroligin-2, suggesting initiation but incomplete differentiation program for inhibitory neurons. This aligns with recent finding that NGN2 generates heterogeneous populations with partial subtype specification (Ang et al., 2024). Therefore, more refined programming strategies are needed to generate fully specified neuronal subtypes from hiPSCs, a goal to which our RLM strategy may meaningfully contribute.

In conclusion, our study demonstrates that UD-proteomics-based RLM strategy significantly enhances health and maturation of NGN2-induced neurons compared to traditional methods. This supports earlier research that NGN2 neurons often exhibit low synaptic activity and demonstrates that tailored neurotrophic support can alleviate this issue. Furthermore, our results suggest that incorporating proteomics-based optimization could enhance application of any hiPSC-derived cell types. These advancements hold significant implications for boosting the reliability of hiPSC-based models in personalized disease research and drug testing, underscoring the critical need for continued refinement of differentiation protocols.

METHODS

The use of human iPSC lines in this study was approved by the Okinawa Institute of Science and Technology Institutional Review Board or Ethics Committee (approval number HSR-2022-012-3), in compliance with relevant regulations.

Cell culture of iPSCs and differentiation into neurons

Human iPSC lines HPS0331 and HPS0328 (Riken BRC Cell Bank, Japan) were maintained in StemFit medium (Ajinomoto, Japan) on iMatrix-511 (Nippi, Japan)-coated 35-mm dishes (Falcon, USA). Neuronal differentiation was initiated as previously described (Zhang et al., 2013). After replating, neurons were cultured in Neurobasal Plus medium supplemented with B27 Plus, 1 μg/mL doxycycline, and 1 μg/mL mouse laminin. Cultures were treated with various combinations of growth factors (BDNF, NT-3, GDNF, CNTF, NRTN, and/or PSPN [PeproTech (USA]), each applied at a concentration of 50 ng/mL, as indicated.

Autaptic iN cell culture

IPSCs were derived from the cell line BIHi005-A, provided by the Berlin Institute of Health Stem cell Core Facility. Autaptic iNs were produced as described previously (Fenske et al., 2019). In brief, 35–45 days after NGN2 induction, iN cultures were washed twice with 0.5 mM EDTA/PBS (Thermo Fisher, USA) and dissociated with Accutase (~30 min at 37°C). Cells were seeded at a density of 4×10^3 cells/35 mm diameter well onto astrocyte microislands to obtain autaptic cultures. Astrocytic islands were produced as described (Fenske et al., 2019). iN microisland cultures were maintained for another 14–21 days (37°C and 5% CO_2) before electrophysiological experiments were performed.

Immunofluorescence imaging

Cells were fixed with 4% PFA in PBS, permeabilized with 0.1% Triton X-100 in PBS for 5 min, and blocked with 10% NGS in PBS for 1 h at room temperature (RT). Primary antibodies were diluted in 5% NGS in PBS, incubated overnight at 4°C, and washed. Secondary antibodies were diluted in wash buffer with 5% NGS, incubated for 1 h at RT, and washed. Imaging was performed on an LSM 780 confocal microscope (Zeiss, Germany) and analyzed with ImageJ. Primary antibodies included anti-Oct4, anti-Ssea4, anti-alpha-tubulin, anti-beta3-tubulin (Abcam, USA), anti-synaptophysin, and anti-active zone (Synaptic Systems, Germany). Secondary antibodies were Alexa Plus 488 and Alexa Plus 555 (Thermo Fisher, USA).

Protein extraction and immunoblotting characterization

Proteins were extracted and quantified as described previously (Taoufiq et al., 2020). Primary antibodies anti-Histone H4 (Abcam, USA), anti-VGLUT1, anti-synaptobrevin 2, and anti-SHANK2 (Synaptic Systems, Germany) were used at 1:1,000. Secondary antibodies goat anti-rabbit, goat anti-mouse, or goat anti-guinea pig HRP conjugates were used at dilution 1:2,000.

Proteomics sample preparation and mass spectrometry identification

Proteomics samples were prepared following the previously described protocol (Taoufiq et al., 2020). Protein and peptide identifications were conducted using Proteome Discoverer v3.0 (Thermo Scientific, USA), with UniprotKB Homo sapiens database queries (Proteome ID: UP000005640), using 10 ppm precursor and 0.6 Da fragment ion tolerances, trypsin specificity, and allowance for up to two missed cleavages. Cysteine carbamidomethylation was set as a fixed modification, with methionine oxidation, asparagine and glutamine deamidation, and N-terminal acetylation as variable modifications. Peptide FDR was controlled at 1% via Percolator in Proteome Discoverer. Quantitative analysis utilized iBAQ values for statistical analyses and volcano plots in Perseus, and UniProtKB's human membrane protein list (Organism code: 9606) was filtered against our dataset in Excel.

Electrophysiology

Mass culture

Whole-cell recordings were made using an EPC-10 USB patch-clamp amplifier (HEKA Elektronik) to measure postsynaptic response in human iPSC-derived neurons, identified with a 60X or 40X water immersion objective on an upright microscope. The composition of the bath solution (mM) was as follows: 125 NaCl, 2.5 KCl, 26 NaHCO3, 1.25 NaHPO4, 10 glucose, 3 myo-inositol, 2 Na pyruvate, 0.5 Na ascorbate (bubbled with 95% O_2 and 5% CO_2), 10 μM picrotoxin, and 0.5 μM strychnine to block GABA and glycine receptors. The composition of the pipette solution (mM) was as follows: 100 Cs methanesulfonate, 30 CsCl, 5 EGTA, 1 MgCl2, 5 QX314-Cl, 10 HEPES, pH 7.3–7.4, and 300–310 mOsm. Neurons were voltage-clamped at −50 mV and EPSCs evoked at 0.05 Hz by a theta-shaped bipolar electrode. Data were acquired at 50 kHz with Patchmaster software, pipette resistance of 5–7 MΩ, series resistance of 14–20 MΩ, and compensated by 70% to reach 7 MΩ.

Autaptic culture

Whole-cell patch-clamp recordings were performed between days 49 and 59 post-induction at RT using a Multiclamp 700B amplifier (Molecular Devices), with series resistance compensated by 70% and analyzed only if < 12 MΩ. Data were acquired at 10 kHz via pClamp 10 software and filtered at 3 kHz. Analysis was done offline with AxoGraph X. The pipette solution contained (mM) 136 KCl, 17.8 HEPES, 1 EGTA, 0.6 MgCl2, 4 ATP-Na2, 0.3 GTP-Na, 12 phosphocreatine, and 50 units/mL phosphocreatine kinase (300 mOsm, pH 7.4). The extracellular solution included NaCl, KCl, HEPES, CaCl2, MgCl2, and glucose (pH 7.3, 300 mOsm). EPSCs were evoked by 2 ms somatic depolarization, and mEPSCs were detected in AxoGraph X, with false positives corrected using NBQX (3 μM).

Synaptosome purification from iPSC neurons

All steps were performed at 4°C using glass tubing for small-scale synaptosomal preparations (Taoufiq et al., 2020). Cultures were harvested by scraping and resuspended in ice-cold sucrose buffer (0.32M sucrose, 4 mM HEPES NaOH, pH 7.4, 1 μg/mL pepstatin, 0.2 mM PMSF). Homogenates were centrifuged 10 min at 800 g (P1 fraction). Supernatants (S1) were centrifuged 15 min at 9,200 g and then 15 min at 10,200 g. The resulting synaptosomal pellets (P2) were stored at −80°C. Synaptosome quality and integrity were assessed by western blot and electron microscopy as described previously (Taoufiq et al., 2020).

Transcriptomics

Total RNA was extracted from iN cells cultured with BN or BCGNr using the NucleoSpin RNA Plus XS kit (Macherey-Nagel) according to the manufacturer's instructions. Library preparation was performed using the KAPA HyperPrep Kit (Roche). Samples were then sent to FASMAC Co., Ltd. (Kanagawa, Japan), where sequencing was conducted following their standard protocols. Sequencing data were analyzed by FASMAC using CLC Genomics Workbench 25 (Qiagen). For normalization of mRNA levels, we selected eight astrocytic genes, as their expression is not expected to change under our culture conditions. We also included the iPSC marker gene *SOX2*, which reflects residual undifferentiated iPSCs in the NGN2 induction protocol. Neuronal genes were not used for normalization since their expression is expected to vary with changes in medium composition. To ensure robustness, we selected reference genes spanning a wide expression range, from lowest (*FGFR2*) to highest (*GFAP*) abundance.

Statistical analysis

Statistical analyses were performed using either the Mann-Whitney U test or two-sample t test, as appropriate. Analyses were conducted using the *pandas*, *scipy*, *seaborn*, *matplotlib*, and *statistics* Python libraries. A p value of <0.05 was considered statistically significant. Exact p values are reported in all figure panels.

RESOURCE AVAILABILITY

Lead contact

Further information and requests for resources and reagents should be directed to and will be fulfilled by the lead contact, Zacharie Taoufiq (zacharie.taoufiq@oist.jp).

Materials availability

Further information and requests may be directed to, and will be fulfilled by, the corresponding authors.

Data and code availability

- The Raw LC-MS/MS data and EXCEL resource table have been deposited to the ProteomeXchange Consortiumvia jPOST with the dataset identifier JPST003435 (PXD057231).
- The raw RNA sequencing data have been deposited in the NCBI Sequence Read Archive (SRA) under accession number (SRR34629791), linked to BioProject (PRJNA1293838) and BioSample accession numbers (SAMN50012407, SAMN500 12408, SAMN50012409, SAMN50012410).

ACKNOWLEDGMENTS

This work was supported by funding from the OIST Proof of Concept Program and grants from JSPS KAKENHI (21K07554) to Z.T., JSPS KAKENHI (20K07771) to D.D., JST COI-NEXT (JPMJPF2205) to Y.G., and a grant from the German Research council (437833719) and OIST scheme of Distinguished Visiting Scholar Program to C.R. We are grateful for the help and support provided by Dr. Y. Hirao from the Instrumental Analysis section of OIST Core Facilities and Marina Khandarkhaeva, Saori Araki, and Asmaa Yahia for technical assistance. We thank the Charité viral core facility for support.

AUTHOR CONTRIBUTIONS

Conceptualization and methodology, D.D. and Z.T.; investigation, D.D., Y.L., T.H., Z.T.; writing—review & editing, D.D., Y.G., C.R., and Z.T.; supervision & funding acquisition, C.R. and Z.T.

DECLARATION OF INTERESTS

D.D. and Z.T. are inventors on a patent application (Patent Application WO2023100949A1) related to the findings presented in this manuscript.

SUPPLEMENTAL INFORMATION

Supplemental information can be found online at https://doi.org/10.1016/j.stemcr.2025.102604.

Received: November 20, 2024
Revised: July 22, 2025
Accepted: July 23, 2025
Published: August 21, 2025

REFERENCES

Ang, C.E., Olmos, V.H., Vodehnal, K., Zhou, B., Lee, Q.Y., Sinha, R., Narayanaswamy, A., Mall, M., Chesnov, K., Dominicus, C.S., et al. (2024). Generation of human excitatory forebrain neurons by cooperative binding of proneural NGN2 and homeobox factor EMX1. Proc. Natl. Acad. Sci. USA *121*, e2308401121.

Bardy, C., van den Hurk, M., Eames, T., Marchand, C., Hernandez, R.V., Kellogg, M., Gorris, M., Galet, B., Palomares, V., Brown, J., et al. (2015). Neuronal medium that supports basic synaptic functions and activity of human neurons in vitro. Proc. Natl. Acad. Sci. USA *112*, E2725–E2734.

Bullmann, T., Kaas, T., Ritzau-Jost, A., Wöhner, A., Kirmann, T., Rizalar, F.S., Holzer, M., Nerlich, J., Puchkov, D., Geis, C., et al. (2024). Human iPSC-Derived Neurons with Reliable Synapses and Large Presynaptic Action Potentials. J. Neurosci. *44*, e0971232024.

Burlingham, S.R., Wong, N.F., Peterkin, L., Lubow, L., Dos Santos Passos, C., Benner, O., Ghebrial, M., Cast, T.P., Xu-Friedman, M. A., Südhof, T.C., and Chanda, S. (2022). Induction of synapse formation by de novo neurotransmitter synthesis. Nat. Commun. *13*, 3060.

Chen, Y., Stevens, B., Chang, J., Milbrandt, J., Barres, B.A., and Hell, J.W. (2008). NS21: re-defined and modified supplement B27 for neuronal cultures. J. Neurosci. Methods *171*, 239–247.

Fenske, P., Grauel, M.K., Brockmann, M.M., Dorrn, A.L., Trimbuch, T., and Rosenmund, C. (2019). Autaptic cultures of human induced neurons as a versatile platform for studying synaptic function and neuronal morphology. Sci. Rep. *9*, 4890.

Giacomelli, E., Vahsen, B.F., Calder, E.L., Xu, Y., Scaber, J., Gray, E., Dafinca, R., Talbot, K., and Studer, L. (2022). Human stem cell models of neurodegeneration: From basic science of amyotrophic lateral sclerosis to clinical translation. Cell Stem Cell *29*, 11–35.

Gunhanlar, N., Shpak, G., van der Kroeg, M., Gouty-Colomer, L.A., Munshi, S.T., Lendemeijer, B., Ghazvini, M., Dupont, C., Hoogendijk, W.J.G., Gribnau, J., et al. (2018). A simplified protocol for differentiation of electrophysiologically mature neuronal networks from human induced pluripotent stem cells. Mol. Psychiatry *23*, 1336–1344.

Huang, Y.A., Zhou, B., Wernig, M., and Sudhof, T.C. (2017). ApoE2, ApoE3, and ApoE4 Differentially Stimulate APP Transcription and Abeta Secretion. Cell *168*, 427–441.e1.

Hulme, A.J., Maksour, S., St-Clair Glover, M., Miellet, S., and Dottori, M. (2022). Making neurons, made easy: The use of Neurogenin-2 in neuronal differentiation. Stem Cell Rep. *17*, 14–34.

Johnson, M.A., Weick, J.P., Pearce, R.A., and Zhang, S.C. (2007). Functional neural development from human embryonic stem cells: accelerated synaptic activity via astrocyte coculture. J. Neurosci. *27*, 3069–3077.

Li, J., Tran, O.T., Crowley, T.B., Moore, T.M., Zackai, E.H., Emanuel, B.S., McDonald-McGinn, D.M., Gur, R.E., Wallace, D.C., and Anderson, S.A. (2021). Association of Mitochondrial Biogenesis With Variable Penetrance of Schizophrenia. JAMA Psychiatry *78*, 911–921.

Pasca, A.M., Sloan, S.A., Clarke, L.E., Tian, Y., Makinson, C.D., Huber, N., Kim, C.H., Park, J.Y., O'Rourke, N.A., Nguyen, K.D., et al. (2015). Functional cortical neurons and astrocytes from human pluripotent stem cells in 3D culture. Nat. Methods *12*, 671–678.

Rhee, H.J., Shaib, A.H., Rehbach, K., Lee, C., Seif, P., Thomas, C., Gideons, E., Guenther, A., Krutenko, T., Hebisch, M., et al. (2019). An Autaptic Culture System for Standardized Analyses of iPSC-Derived Human Neurons. Cell Rep. *27*, 2212–2228.e7.

Rowe, R.G., and Daley, G.Q. (2019). Induced pluripotent stem cells in disease modelling and drug discovery. Nat. Rev. Genet. *20*, 377–388.

Shi, Y., Inoue, H., Wu, J.C., and Yamanaka, S. (2017). Induced pluripotent stem cell technology: a decade of progress. Nat. Rev. Drug Discov. *16*, 115–130.

Takahashi, K., and Yamanaka, S. (2006). Induction of pluripotent stem cells from mouse embryonic and adult fibroblast cultures by defined factors. Cell *126*, 663–676.

Taoufiq, Z., Ninov, M., Villar-Briones, A., Wang, H.Y., Sasaki, T., Roy, M.C., Beauchain, F., Mori, Y., Yoshida, T., Takamori, S., et al. (2020). Hidden proteome of synaptic vesicles in the mammalian brain. Proc. Natl. Acad. Sci. USA *117*, 33586–33596.

Zhang, Y., Pak, C., Han, Y., Ahlenius, H., Zhang, Z., Chanda, S., Marro, S., Patzke, C., Acuna, C., Covy, J., et al. (2013). Rapid single-step induction of functional neurons from human pluripotent stem cells. Neuron *78*, 785–798.

Stem Cell Reports, Volume *20*

Supplemental Information

Proteomics-based receptor-ligand matching enhances differentiation maturity of human-stem-cell-derived neurons

Dimitar Dimitrov, Yi Lien, Tetsuya Hori, Yukiko Goda, Christian Rosenmund, and Zacharie Taoufiq

Proteomics-based Receptor-ligand Matching Enhances Differentiation Maturity of Human Stem Cell-derived Neurons

SUPPLEMENTAL INFORMATION

Figure S1: Distinct Synaptosomal UD Proteome Profiles of NGN2-Neurons Versus Mammalian Whole Brain.

(A) Electron microscopy of iN synaptosomal fraction showing intact synaptic components, such as the active zone (AZ) and synaptic vesicles (SVs).

(B) Immunoblot analysis of iN subcellular fractionation showing significant purity of iN synaptosomes (P2) from the enrichment of synaptic proteins.

C) Comparison of synaptic protein families (synaptotagmins, syntaxins, active zone and postsynaptic density proteins) identified by HD vs UD proteomics.

(D) Numbers of identified proteins in iN synaptosomes by HD vs UD proteomics.

(E) Quantitative UD proteomic comparison of synaptic machinery in iN synaptosomes versus mammalian whole brain purified synaptosomes. Bar graphs represent normalized iBAQ ratios of proteins (mean ratio ± SEM, n = 3; p-value: ***< 0.001).

(F) Schematic diagram showing major differences in the synaptosomal proteome between NGN2-induced neurons from hiPSCs and those from the mammalian (rat) whole brain. NGN2 synapses are depicted as 100% glutamatergic due to the incomplete and low expression of key inhibitory synapse machinery such as VGAT.

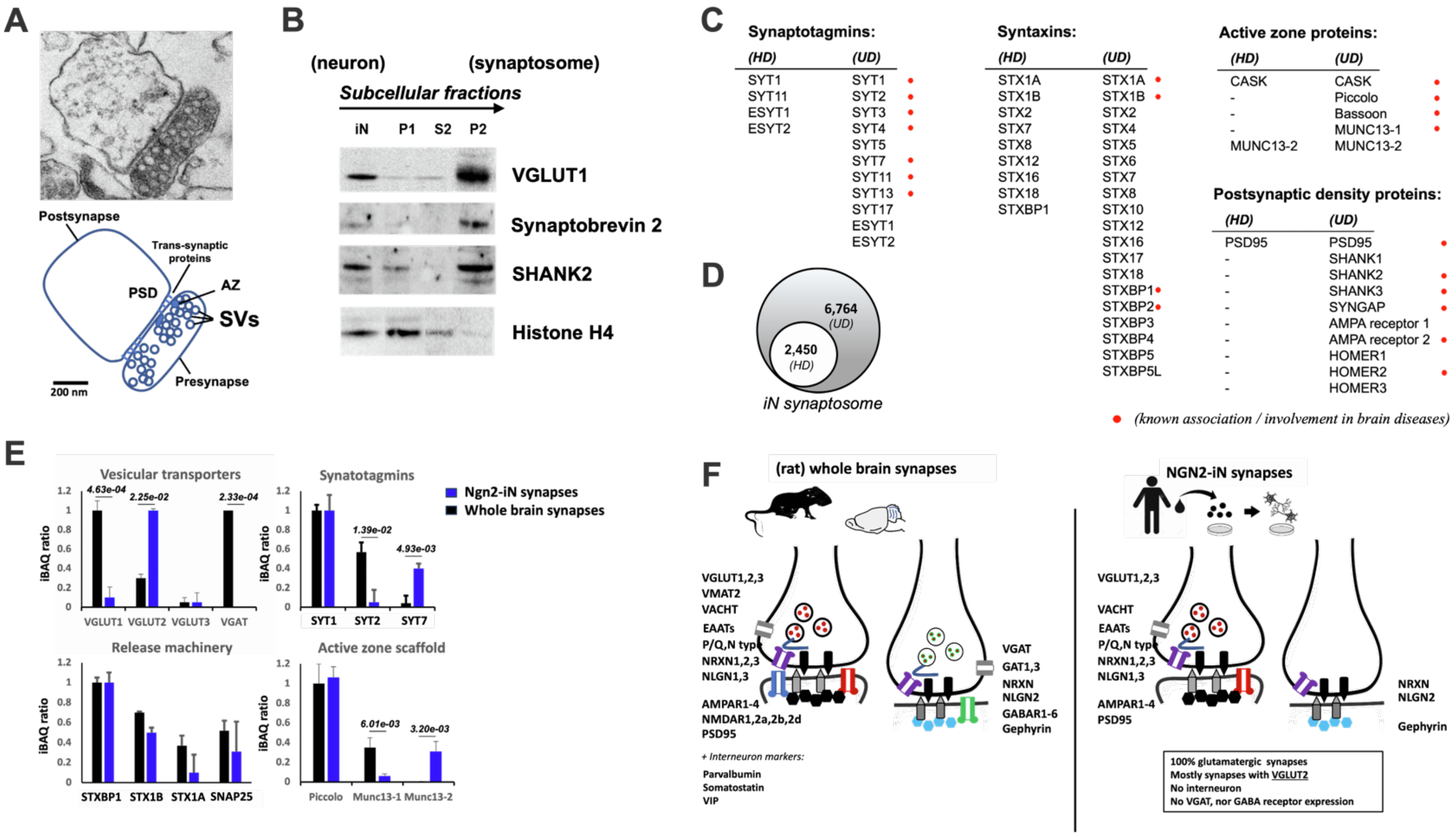

Figure S2: Transcriptomic Analysis of BN vs. BCGNr iN Cells Reveals Upregulation of Neuronal Structural and Immune Stress-Resistance Genes in BCGNr Neurons.

(A) For normalization of BN and BCGNr cell whole transcriptomes, eight astrocytic genes and one iPSC gene were selected as reference genes, covering a broad range of expression levels, as their expression is not expected to change under our culture conditions. The scaling factor was derived from the geometric mean of their expression. Scatter plots and correlation coefficients are shown before and after correction and normalization.

(B) Heatmap showing fold changes in mRNA expression in BCGNr vs BN iN cells. BCGNr cells show higher expression of synaptic (e.g., *SYP, SYN1, GRIA1*), structural (e.g., *TUBB3, MAP2, MAPT*), and neuronal activation markers (*FOS, EGR1*), while astrocytic and iPSC-related genes (e.g., *GFAP, GJA1, SOX2*) remain unchanged or slightly lower than in BN cells (cell differentiation batches n = 2).

(C) Identification of the most strongly differentially expressed genes between BN and BCGNr iN cells. In BCGNr, *DTX3L, SAMD9*, and IFI6 showed 800-, 180-, and 100-fold higher transcript counts, respectively. Conversely, *AMIGO3, 7SK_5*, and *OPRK1* were highly downregulated in BCGNr compared to BN iN cells by 1000-, 300-, and 15-fold (cell differentiation batches n = 2).

(D) Table summarizing the most strongly differentially expressed genes and their associated functions. Genes upregulated in BCGNr cells remain unstudied in neurons but are associated with cell survival, stress resistance, and suppression of mitochondrial stress-related during immune responses. In contrast, genes upregulated in BN cells are well-characterized in neurons and are associated with inhibition of axon and neurite growth, delayed differentiation, and altered neuronal excitability.

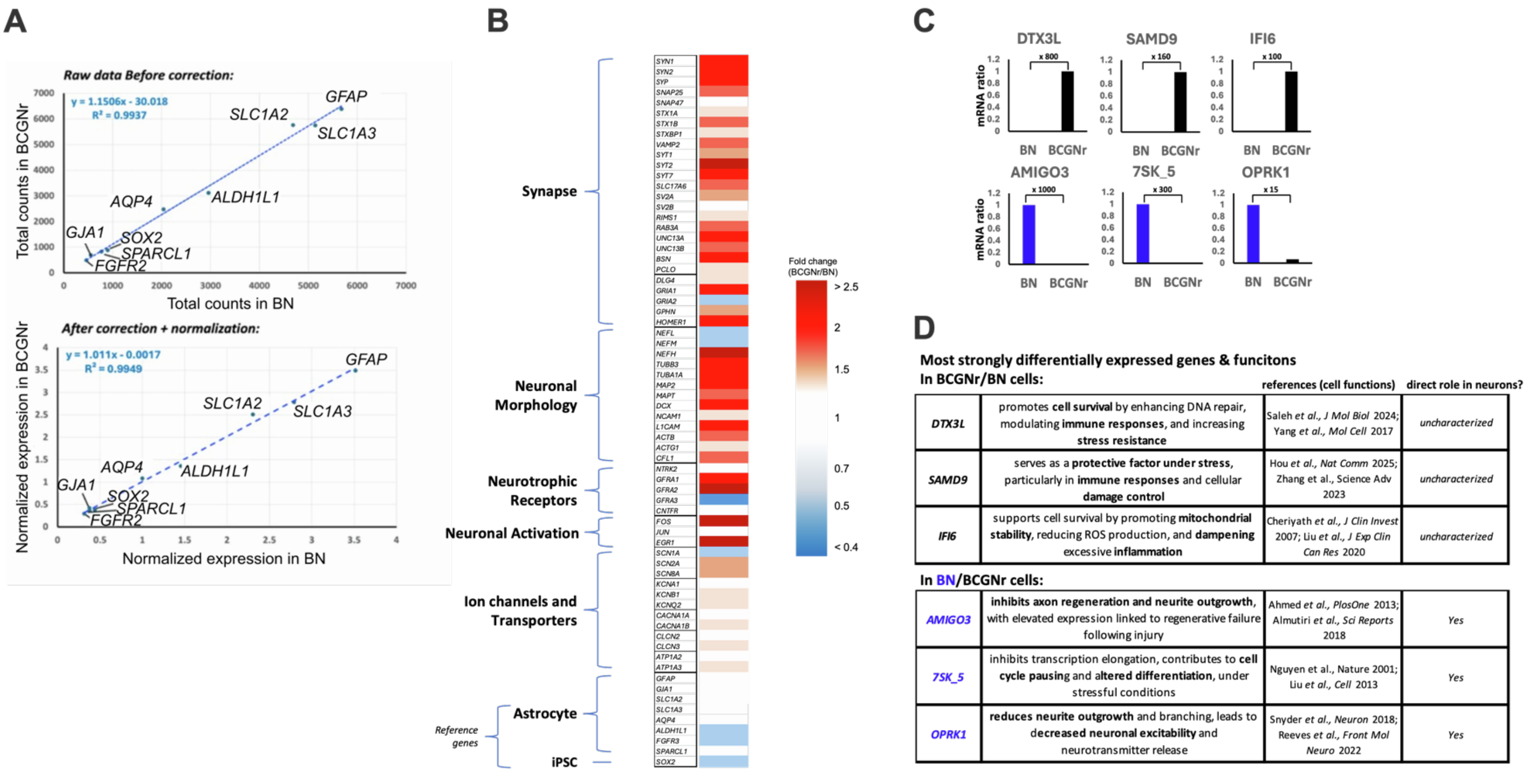

Most strongly differentially expressed genes & funcitons

In BCGNr/BN cells:

		references (cell functions)	direct role in neurons?
DTX3L	promotes **cell survival** by enhancing DNA repair, modulating **immune responses**, and increasing **stress resistance**	Saleh *et al., J Mol Biol* 2024; Yang *et al., Mol Cell* 2017	*uncharacterized*
SAMD9	serves as a **protective factor under stress**, particularly in **immune responses** and cellular **damage control**	Hou *et al., Nat Comm* 2025; Zhang et al., Science Adv 2023	*uncharacterized*
IFI6	supports cell survival by promoting **mitochondrial stability**, reducing ROS production, and **dampening** excessive **inflammation**	Cheriyath *et al., J Clin Invest* 2007; Liu *et al., J Exp Clin Can Res* 2020	*uncharacterized*

In BN/BCGNr cells:

AMIGO3	**inhibits axon regeneration and neurite outgrowth**, with elevated expression linked to regenerative failure following injury	Ahmed *et al., PlosOne* 2013; Almutiri *et al., Sci Reports* 2018	*Yes*
7SK_5	inhibits transcription elongation, contributes to **cell cycle pausing** and **altered differentiation**, under stressful conditions	Nguyen et al., Nature 2001; Liu *et al., Cell* 2013	*Yes*
OPRK1	**reduces neurite outgrowth** and branching, leads to **decreased neuronal excitability** and neurotransmitter release	Snyder *et al., Neuron* 2018; Reeves *et al., Front Mol Neuro* 2022	*Yes*

Supplemental References:

(Related to Figure S2D)

Ahmed, Z., Douglas, M.R., John, G., Berry, M., and Logan, A. (2013). AMIGO3 is an NgR1/p75 co-receptor signalling axon growth inhibition in the acute phase of adult central nervous system injury. PLoS One *8*, e61878. 10.1371/journal.pone.0061878.

Almutiri, S., Berry, M., Logan, A., and Ahmed, Z. (2018). Non-viral-mediated suppression of AMIGO3 promotes disinhibited NT3-mediated regeneration of spinal cord dorsal column axons. Sci Rep *8*, 10707. 10.1038/s41598-018-29124-z.

Cheriyath, V., Glaser, K.B., Waring, J.F., Baz, R., Hussein, M.A., and Borden, E.C. (2007). G1P3, an IFN-induced survival factor, antagonizes TRAIL-induced apoptosis in human myeloma cells. J Clin Invest *117*, 3107-3117. 10.1172/JCI31122.

Hou, G., Beatty, W., Ren, L., Ooi, Y.S., Son, J., Zhu, Y., Sheng, Q., Huang, W., Li, D., Liu, C., et al. (2025). SAMD9 senses cytosolic double-stranded nucleic acids in epithelial and mesenchymal cells to induce antiviral immunity. Nat Commun *16*, 3756. 10.1038/s41467-025-59090-w.

Liu, W., Ma, Q., Wong, K., Li, W., Ohgi, K., Zhang, J., Aggarwal, A., and Rosenfeld, M.G. (2013). Brd4 and JMJD6-associated anti-pause enhancers in regulation of transcriptional pause release. Cell *155*, 1581-1595. 10.1016/j.cell.2013.10.056.

Liu, Z., Gu, S., Lu, T., Wu, K., Li, L., Dong, C., and Zhou, Y. (2020). IFI6 depletion inhibits esophageal squamous cell carcinoma progression through reactive oxygen species accumulation via mitochondrial dysfunction and endoplasmic reticulum stress. J Exp Clin Cancer Res *39*, 144. 10.1186/s13046-020-01646-3.

Nguyen, V.T., Kiss, T., Michels, A.A., and Bensaude, O. (2001). 7SK small nuclear RNA binds to and inhibits the activity of CDK9/cyclin T complexes. Nature *414*, 322-325. 10.1038/35104581.

Reeves, K.C., Shah, N., Munoz, B., and Atwood, B.K. (2022). Opioid Receptor-Mediated Regulation of Neurotransmission in the Brain. Front Mol Neurosci *15*, 919773. 10.3389/fnmol.2022.919773.

Saleh, H., Liloglou, T., Rigden, D.J., Parsons, J.L., and Grundy, G.J. (2024). KH-like Domains in PARP9/DTX3L and PARP14 Coordinate Protein-Protein Interactions to Promote Cancer Cell Survival. J Mol Biol *436*, 168434. 10.1016/j.jmb.2023.168434.

Snyder, L.M., Chiang, M.C., Loeza-Alcocer, E., Omori, Y., Hachisuka, J., Sheahan, T.D., Gale, J.R., Adelman, P.C., Sypek, E.I., Fulton, S.A., et al. (2018). Kappa Opioid Receptor Distribution and Function in Primary Afferents. Neuron *99*, 1274-1288 e1276. 10.1016/j.neuron.2018.08.044.

Yang, C.S., Jividen, K., Spencer, A., Dworak, N., Ni, L., Oostdyk, L.T., Chatterjee, M., Kusmider, B., Reon, B., Parlak, M., et al. (2017). Ubiquitin Modification by the E3 Ligase/ADP-Ribosyltransferase Dtx3L/Parp9. Mol Cell *66*, 503-516 e505. 10.1016/j.molcel.2017.04.028.

Zhang, F., Ji, Q., Chaturvedi, J., Morales, M., Mao, Y., Meng, X., Dong, L., Deng, J., Qian, S.B., and Xiang, Y. (2023). Human SAMD9 is a poxvirus-activatable anticodon nuclease inhibiting codon-specific protein synthesis. Sci Adv *9*, eadh8502. 10.1126/sciadv.adh8502.
